# Urine neutrophil gelatinase-associated lipocalin concentration in healthy newborns during the first three postnatal days

**DOI:** 10.11613/BM.2020.030706

**Published:** 2020-10-15

**Authors:** Vinka Mikulić, Dunja Rogić, Ivanka Mikulić, Kristina Ljubić, Ana Ćuk, Vajdana Tomić, Helena Radić Mišković

**Affiliations:** 1 Department of Laboratory Diagnostics, University Clinical Hospital Mostar, Mostar, Bosnia and Herzegovina; 2 Department of Laboratory Diagnostics, University Hospital Center Zagreb, Zagreb, Croatia; 3 Department of Obstetrics and Gynaecology, University Clinical Hospital Mostar, Mostar, Bosnia and Herzegovina; 4 Department of Neonatology, University Clinical Hospital Mostar, Mostar, Bosnia and Herzegovina

**Keywords:** neutrophil gelatinase-associated lipocalin, urine, healthy term newborns

## Abstract

**Introduction:**

Urine neutrophil gelatinase-associated lipocalin (uNGAL) is a biochemical marker significant for early prediction of acute kidney injury in adults. However, it has not been examined sufficiently among the infant population, particularly newborns in terms of reference values. The aim of our study was to determine the concentration of uNGAL in healthy term newborns and to determine if there was a difference in uNGAL concentration according to gender, postnatal age and birth weight.

**Materials and methods:**

Our study involved 81 healthy term newborns birth (≥ 37 weeks, Apgar score ≥ 8 in the first minute after birth, CRP < 5 mg/L). Urine NGAL was measured using chemiluminescent microparticle immunoassay (CMIA) within 72 hours after birth, on Architect plus ci8200 analyser (Abbott, Chicago, USA). Data were analysed using Statistica software.

**Results:**

The median concentration of uNGAL in the whole study group of healthy term newborns was 27.1 ng/mL (16.5-56.0 ng/mL) (newborn girls, 27.1 ng/mL (15.8-47.9 ng/mL); newborn boys, 27.9 ng/mL (16.5-61.0 ng/mL), P = 0.941). Median uNGAL concentration according to postnatal age expressed in days was 28.2 ng/mL (11.7-57.2 ng/mL) 1^st^ day, 28.9 ng/mL (16.5-64.2 ng/mL) 2^nd^ day and 23.9 ng/mL (20.2-46.6) 3^rd^ day, P = 0.863. Regarding birth weight for newborns < 3500 g, median concentration was 25.0 ng/mL (16.5-45.4 ng/mL) and for weight ≥ 3500 g 30.6 ng/mL (16.5-64.2 ng/mL), P = 0.455.

**Conclusions:**

There were no significant difference in uNGAL concentration in relation to gender, postnatal age and birth weight.

## Introduction

Neutrophil gelatinase-associated lipocalin (NGAL), also known as lipocalin-2, LCN2, siderocalin, 24p3, is a 25 kDa molecular weight protein. It belongs to the lipocalin superfamily, which consists of proteins that transport small hydrophobic molecules such as lipids, steroid hormones and retinoids (*1*). This biochemical marker appears to be significant in early detection of acute kidney injury, but currently it has not been sufficiently examined in the paediatric population, particularly in newborns population. Although NGAL can be secreted by different types of cells, some research data indicate its high diagnostic sensitivity and specificity in acute kidney injury. Numerous studies emphasise its predictive, diagnostic and prognostic benefits in acute kidney injury (*2*-*8*). To date, research on urine NGAL in newborn population has included certain conditions and illnesses such as asphyxia and sepsis, which might cause acute kidney injury. In most research, reference groups consisted of a small number of healthy newborns (9-12). There is not enough data about NGAL concentration in healthy infants, including both serum and urine NGAL, which challenges the probability of correct interpretation in different pathological conditions. Thus, the aim of the study was to determine the concentration of urine NGAL (uNGAL) in healthy term newborns, as well as to determine if there was a difference between urine NGAL concentration in relation to gender, postnatal age and birth weight. The hypothesis was that uNGAL concentration does not depend on gender, postnatal age and birth weight.

## Materials and methods

### Subjects

The present study was prospective and included 81 healthy newborn (newborns girls, N = 31 and newborn boys, N = 50) born at University Hospital Mostar, Bosnia and Herzegovina, from April to October 2019. The study was approved by the Ethics Committee of the University Clinical Hospital Mostar (Ethical approving number 2272/12 at UCHM) in adherence to the Declaration of Helsinki. Written informed consent was obtained from all parents after explaining the purpose of the study. The newborns included in the study were delivered from full-term pregnancies and inclusion criteria after clinical examination were as follows: gestational age at birth ≥ 37 weeks, Apgar score ≥ 8 in the first minute after birth, C-reactive protein (CRP) < 5 mg/L, normal physical and ultrasound examination (*12*-*14*). If routine clinical examination after birth showed the presence of infection, cardiac disease, congenital anomalies and chromosomal disorder, the newborns were excluded from the study.

### Methods

The present study used the leftover serum samples that had previously been analysed in routine laboratory tests (mainly bilirubin) required by a neonatologist after the post-delivery examination. The leftover serum was used to determine CRP concentration, which was the including criteria for the study. Venous blood samples were collected in Micro sample tubes with gel (Sarstedt, Nümbrecht, Germany; volume 1.1 mL) with Micro needles 23 G (Sarstedt, Nümbrecht, Germany). CRP concentration was determined using a CRP Latex reagent kit (Beckman Coulter, Co. Clare, Ireland) on Beckman Coulter Chemistry Analyser AU680 (Beckman Coulter, Brea, USA), controlled two times a day (ITA Control Serum Level 1, REF. ODC0014; ITA Control Serum Level 2, REF.ODC0015; ITA Control Serum Level 3, REF.ODC0016). Urine samples (3 – 5 mL) were obtained within 72 hours after birth in paediatric urine sterile collector for newborns with a birth weight over 2000 g (B. Braun Medical, Saint-Cloud, France) and centrifuged at 400 RCF over five minutes and stored at - 80 °C until analysis. Urine NGAL concentration was quantitatively determined using original manufacturer Architect Urine NGAL reagent kit (REF.^1^P37, LOT.89159UI00, Abbott, Sligo, Ireland) on Architect plus ci8200 analyser (Abbott, Chicago, USA). The Architect Urine NGAL assay is a two-step immunoassay for the quantitative detection of NGAL in human urine using chemiluminescent microparticle immunoassay (CMIA) technology. The Architect urine NGAL assay utilizes a non-competitive, sandwich format with chemiluminescent signal detection. The assay includes a microparticle reagent prepared by covalently attaching an anti-NGAL antibody to paramagnetic particles and a conjugate reagent prepared by labelling a second anti-NGAL antibody with acridinium. Following the immunochemistry steps, the solid phase is washed again and the acridinium label is triggered with peroxide and base to generate the signal (*15*). The assay was calibrated according to the manufacturer’s instructions (Architect Urine NGAL Calibrators, Abbott, Ireland REF.^1^P37-01, LOT.86303UI00, 0.0 ng/mL, 10.0 ng/mL, 100.0 ng/mL, 500.0 ng/mL, 1000.0 ng/mL, 1500.0 ng/mL) and checked by using commercial controls (Architect Urine NGAL Controls, Abbott, Ireland, REF.^1^P37-10, LOT.90414UI00, Low Control 20.0 ng/mL, Medium Control 200.0 ng/mL, High Control 1200.0 ng/mL). According to Architect Urine NGAL manufacturer’s study, which was based on Laboratory Standards Institute (CLSI) EP15-A2 guidance, CV was ≤ 6.7%.

### Statistical analysis

Data analysis was performed using Statistica 12.0. software (StatSoft, Tulsa, OK, USA). Non-parametric tests were used because the obtained data were not normally distributed (Shapiro Wilk test). Measurement data were indicated as median and interquartile range (from first (Q1) to the third quartile (Q3)), except for gestation age that is presented as median and minimum – maximum. To compare two groups, Mann-Whitney *U*-test was used. Kruskal-Wallis test was used to compare more groups. P values < 0.05 were considered statistically significant.

## Results

The study comprised 81 healthy term neonates, newborn girls (subgroup F) and newborn boys (subgroup M). The respective median gestational ages were 39 (37-41) weeks in females, and 40 (37-41) weeks in males. Median values with an interquartile range along with general characteristics of healthy newborns (birth weight, birth length and Apgar score) differentiated by gender are shown in Table 1. No parameter was shown to be statistically significantly different between genders, although we observed an almost significant difference regarding birth weight (P = 0.056). Median uNGAL concentration in the whole study group was 27.1 ng/mL (16.5-56.0 ng/mL). The values did not differ between genders (P = 0.941), in relation to postnatal age (P = 0.863) nor in relation to birth weight (P = 0.455) (Table 2).

**Table 1:** General characteristics of healthy newborns

**Characteristic**	**F (N = 31)**	**M (N = 50)**	P value*
Gestation age (weeks)	39.4 (37.0-41.1)	39.6 (37.0-41.4)	0.872
Birth weight (g)	3350 (2950-3600)	3525 (3250-3800)	0.056
Birth length (cm)	54 (53-56)	55 (53-56)	0.134
Apgar score (1 min)	10 (10-10)	10 (10-10)	0.741
Data are presented as the median and interquartile range, except for gestation age that is presented as median and minimum – maximum. *Mann-Whitney U-test. F – newborn girls. M – newborn boys. P < 0.05 was considered statistically significant.			

**Table 2:** Urine neutrophil gelatinase-associated lipocalin (uNGAL) concentration of healthy newborns

**Parameter**	**uNGAL (ng/mL)**	**P**
Total group (N = 81)	27.1 (16.5-56.0)	/
Gender		
F (N = 31)	27.1 (15.8-47.9)	0.941*
M (N = 50)	27.9 (16.5-61.0)	
Postnatal age		
1st day (N = 20)	28.2 (11.7-57.2)	0.863^†^
2nd day (N = 51)	28.9 (16.5-64.2)	
3rd day (N = 10)	23.9 (20.2-46.6)	
Birth weight		
< 3500 g (N = 41)	25.0 (16.5-45.4)	0.455*
≥ 3500 g (N = 40)	30.6 (16.5-64.2)	
Data are presented as the median and interquartile range. *Mann-Whitney U-test. ^†^Kruskal-Wallis test. P<0.05 was considered statistically significant.		

## Discussion

The study demonstrated that there was no significant difference in uNGAL concentration in relation to gender, postnatal age and birth weight, which supports the hypothesis mentioned beforehand. There have been several studies that included uNGAL concentration measurement in a healthy newborn population. One of these studies, done by Chen *et al.*, included measurement of uNGAL in 38 term newborns when they were three days old. The median concentration of uNGAL was 88.1 ng/mL (*16*). The uNGAL values were higher than in our study. Another study by Sarafidis *et al.* was carried out on 22 term infants on their first and third day of life. Median urine NGAL concentration on the first day of a newborns life was 6.8 ng/mL, while this concentration was 7.1 ng/mL on the third day (*17*). Abdelhady *et al.* carried out a research on a control group of 20 newborns and results showed the median uNGAL concentration of 9 ng/mL (*18*). The only research carried out on a higher number of subjects was conducted by Kamianowska *et al*. (*19*). This research included 88 term-neonates with median uNGAL concentration of 16.74 ng/mL. In contrast to our study, their study proved that uNGAL concentration is significantly higher in female newborns. The median concentration of uNGAL was 24.16 ng/mL for female neonates and 13.30 ng/mL for male neonates, P < 0.01. The uNGAL values in these studies were lower than those obtained by us (*17*-*19*). The reasons for the reported differences may result from different measurement methods, different manufacture reagents and antibodies. Study by Krzeminska *et al*. on 30 healthy adults demonstrated that uNGAL concentration obtained by CMIA (Abbott) method was significantly higher than ELISA (R&D Systems) (*20*). It is highly important to note that the concentrations in the present study were measured using a fully standardized and automated CMIA method, as opposed to most above-mentioned studies using ELISA method from different manufacturers (*16*-*19*). Similar results to the present study were published by Cangemi *et al.* on 25 newborns and the median concentration of uNGAL was 30.3 ng/mL (CMIA method, Abbott). Also in their study, there wasn´t a significant difference regarding uNGAL value associated with gender (*21*). Early research, which examined a possible correlation between uNGAL, birth weight and postnatal age had obtained conflicting results. Two separate studies, done by Suchojad *et al.* and Lavery *et al.*, showed a negative correlation between uNGAL and birth weight (*22*, *23*). On the contrary, our results are in concordance with Elmas *et al.* that showed no differences in uNGAL concentrations in regard to birth weight (*24*). The study Seaidi *et al.* showed that postnatal age is associated with uNGAL concentration (*25*). On the other hand, our results agree with Lavery *et al.* study that showed no differences in uNGAL concentration regarding postnatal age (*23*). There is a relatively small number of studies that deal with uNGAL concentration measurement in healthy children, especially in newborns within the first days of life. Moreover, other published papers addressing uNGAL concentration measurement include small groups of subjects or children of different ages. Based on the literature review, the present study is the first one that used automated CMIA method for measurement of urine NGAL concentration over 80 healthy term neonates. The big advantage of CMIA method is the shorter time of measurement of uNGAL (in our case about 35 minutes) which is highly important for routine clinical use, and is less demanding in technical manner. Our findings pointed towards a usable reference range limits for uNGAL among the newborn population. However, similar to all previous studies, our study had the same potential limitation - we did not use uNGAL/creatinine ratio. Our rationale was that at this early stage of life creatinine excretion is not constant and therefore cannot be used to improve the variability of the results due to urine concentration. In conclusion, since it was carried out in healthy new-borns, as expected, the present study showed no significant difference in concentration of uNGAL in relation to gender, postnatal age and birth weight of new-borns. Future studies should include a higher number of neonates utilizing the same standardized method in order to precisely determine NGAL reference interval for neonate population and thus contribute to the possibility of early acute kidney injury recognition in the first days of life.
